# Generation of Vestibular Tissue-Like Organoids From Human Pluripotent Stem Cells Using the Rotary Cell Culture System

**DOI:** 10.3389/fcell.2019.00025

**Published:** 2019-03-05

**Authors:** Cristiana Mattei, Rebecca Lim, Hannah Drury, Babak Nasr, Zihui Li, Melissa A. Tadros, Giovanna M. D'Abaco, Kathryn S. Stok, Bryony A. Nayagam, Mirella Dottori

**Affiliations:** ^1^Centre for Neural Engineering, Melbourne School of Engineering, The University of Melbourne, Melbourne, VIC, Australia; ^2^Department of Biomedical Engineering, Melbourne School of Engineering, The University of Melbourne, Melbourne, VIC, Australia; ^3^School of Biomedical Sciences and Pharmacy, Faculty of Health and Medicine, University of Newcastle, Newcastle, NSW, Australia; ^4^Department of Electrical and Electronic Engineering, Melbourne School of Engineering, The University of Melbourne, Melbourne, VIC, Australia; ^5^ARC Centre of Excellence for Integrative Brain Function, The University of Melbourne, Melbourne, VIC, Australia; ^6^Departments of Audiology and Speech Pathology and Department of Medical Bionics, The University of Melbourne, Melbourne, VIC, Australia; ^7^Illawarra Health and Medical Research Institute, University of Wollongong, Wollongong, NSW, Australia

**Keywords:** vestibular hair cells, human pluripotent stem cells, organoids, human fetal tissue, inner ear

## Abstract

Hair cells are specialized mechanosensitive cells responsible for mediating balance and hearing within the inner ear. In mammals, hair cells are limited in number and do not regenerate. Human pluripotent stem cells (hPSCs) provide a valuable source for deriving human hair cells to study their development and design therapies to treat and/or prevent their degeneration. In this study we used a dynamic 3D Rotary Cell Culture System (RCCS) for deriving inner ear organoids from hPSCs. We show RCCS-derived organoids recapitulate stages of inner ear development and give rise to an enriched population of hair cells displaying vestibular-like morphological and physiological phenotypes, which resemble developing human fetal inner ear hair cells as well as the presence of accessory otoconia-like structures. These results show that hPSC-derived organoids can generate complex inner ear structural features and be a resource to study inner ear development.

## Introduction

Each human inner ear contains ~90,000 sensory hair cells that convert sound and motion from mechanical energy to electrical signals within the mammalian cochlea (hearing) and balance (vestibular) organs, respectively. Inner ear hair cells are exquisitely sensitive, and this feature also renders hair cells prone to damage, disease, and vulnerable to aging. Once damaged, human inner ear hair cells do not regenerate and their destruction can ultimately lead to loss of hearing and/or balance.

Inner ear hair cells of the auditory and vestibular systems are structurally similar: all have hair bundles or stereocilia that emanate from their apical surface. The mode of activation is also similar: sound waves or head movement causes deflection of these stereocilia resulting in a cascade of ion fluxes and signaling molecules that excites hair cells. However, there is a diversity of hair cell types within the inner ear, each with distinct roles. This suggests there are different signaling molecules, transcription factors, and pathways of differentiation that drive cells to become one of four types; vestibular (type I or type II) hair cells or cochlear (inner or outer) hair cells.

Over the last decade, stem cell biology has advanced significantly, such that human pluripotent stem cells (hPSCs) can be generated from any individual. One of the greatest benefits of this technology is that it provides an opportunity to study human tissue at a cellular level, particularly cell types that would otherwise be inaccessible and/or difficult to access in human. Deriving human inner ear hair cell-like cells from pluripotent stem cells has proved surprisingly challenging to date and has only been published by a select few (Ronaghi et al., 2014; Ohnishi et al., 2015; Koehler et al., 2017) and only one describing generation of human inner ear organoids and vestibular hair cells used spinner flasks/orbital shakers (Koehler et al., 2017). All studies are limited by their comparisons to rodent anatomy and physiology.

We have recently developed a three-dimensional organoid cell culture model using rotary cell culture (RCCS) (Mattei et al., 2018). The new model produces large numbers of dorsal hindbrain progenitors, a region from which we know the inner ear sensory hair cells and neurons are derived. Here we describe an alternative method based on the utilization of the RCCS for generating inner ear organoids consisting of an enriched population of inner ear hair cell-like cells, which display key functional properties of human inner ear hair cell phenotypes along with appropriate anatomical features. We show that microgravity-derived organoids consist of cells which are both ATOH1 and myosin VIIa immunoreactive and show kinocilia-like projections surrounded by stereocilia. Importantly, organoid-derived cells are physiologically similar to developing human fetal vestibular hair cells, exhibiting comparable voltage-activated conductance, and exhibit the presence of inner ear-like accessory structures. These findings are significant for establishing a human inner ear *in vitro* model to study development of the vestibular system and also pursue therapies to treat inner ear degeneration.

## Materials and Methods

### Culture and Differentiation of hPSCs

This project is approved by University of Melbourne Human Ethics committee (#1545384 and 1545394). Human ES cell lines, H3 (kindly provided by E. Stanley and A. Elefanty, Murdoch Institute Children Research, Australia) and H9 (WA09, WiCell), and human iPS cell line 007 (Hernández et al., 2016), were maintained as bulk culture in feeder-free conditions on vitronectin (StemCell Technologies) coated dish (Corning) using Tesr-E8 basal medium (StemCell Technologies). For induction, aggregates of 1,000 hPS cells were plated in U-bottom ultra-low attachment 96-multiwell plates (Corning) in Tesr-E8 basal medium to form embryoid bodies. After 24 h, embryoid bodies were transferred into the RCCS (Synthecon) in N2B27 medium containing 1:1 mix of neurobasal (NB) medium with DMEM/F12 medium, 1% insulin/transferrin/selenium, 1% N2 supplement, 1% retinol-free B27 supplement, 1% glutamax, 1% penicillin streptomycin (Life Technologies), 0.3% glucose (Sigma Aldrich), supplemented with inhibitors SB431542 (10 μM, Tocris) and LDN 193189 (100 nM, KareBay Biochem). Medium change was performed on day 3 of induction, replaced with N2B27 medium supplemented with FGF (20 ng/ml, Peprotech) on day 7 and changed on day 10. On day 14 medium change was performed and organoids were cultured with NB medium containing 1% insulin/transferrin/selenium, 1% N2 supplement, 1% retinol-free B27 supplement, 1% glutamax, 1% penicillin streptomycin, supplemented with FGF and EGF (20 ng/ml, Peprotech) up to day 28 and with supplement-free NB medium up to day 56. On day 56 medium change was performed and replaced with supplement-free NB medium and 1:4 DMEM/F12 containing 1% N2 supplement, 1% glutamax and 0.6% glucose. At every medium change the DMEM/F12 concentration was gradually increased by 25%. From day 14 to day 133 medium change was performed every third day. The RCCS was placed in an incubator at 5% CO_2_ and 37°C and speed rate was gradually increased overtime to ensure a continuous falling motion of organoids. Bright field images of organoids were obtained using a ZEISS Observer z1 with ZEN imaging software.

### RCCS Set Up Procedure

At day 1, 300 embryoid bodies were transferred into each RCCS 10 ml-vessel through the sterile valves on the top of the vessel, using a 10 ml syringe as instructed in the manufacturer's operation manual. The RCCS was placed in an incubator at 5% CO_2_ and 37°C. At day 1, the speed rate was 18 RPM as indicated by the tachometer's display on the RCCS power supplier. By day 28 speed rate was increased by 5–6 RPM and up to 30 RPM by day 98 till day 133. Speed rate was gradually increased over time depending on organoid size, to ensure a continuous falling motion of organoids through the medium during vessel rotation and therefore facilitate their exposure to nutrients, as instructed by the manufacturer.

### Immunohistochemistry

Organoids were collected and fixed with 4% paraformaldehyde for 1 h on ice. Fixed samples were incubated overnight at 4°C with 20% sucrose to cryoprotect. Samples were embedded with O.C.T. compound (VWR Chemicals) and sectioned using a cryostat to obtain 12–14 μm sections. For immunostaining, cryosections were permeabilized using 0.2% Triton-X100 solution and incubated with primary and secondary antibodies in 10% Fetal Calf Serum (Millipore)/phosphate buffered saline DPBS (Life Technologies) blocking solution. The following primary antibodies were used: anti-Tub alpha 4a (mouse, 1:250, Sigma-Aldrich, T6793), anti-Myo7a (rabbit, 1:100, Proteus, 256790), anti-Ctbp2 (mouse, 1:400, BD Transduction Lab, 612044), anti-Pax2 (rabbit, 1:200, BioLegend, 901001), anti-Atoh1 (rabbit, 1:500, Proteintech, 212151AP), anti-PAX7 (mouse, 1:20, DSHB), anti-Pax6 (mouse, 1:80, DSHB), anti-Sox2 (mouse, 1:50, R&D, MAB2018) and anti-TubβIII (mouse, 1:500, Merch, MAB1637). FActin was stained using fluorescein (FITC) phalloidin (1:80, Thermo Fisher Scientific, F432). Alexa Fluor 488 and 568 conjugated anti-mouse IgG and Alexa Fluor 488 and 568 conjugated anti-rabbit IgG were used as secondary antibodies at a final concentration of 1:1000 (Life Technologies). Nuclei were visualized using DAPI counterstain (1 μg/ml final concentration, Sigma-Aldrich). Samples were mounted onto glass slides using moviol mountant followed by image capture using a Nikon A1R confocal microscope or ZEISS AxioObserver z1 fluorescence microscope.

### AM1-44 Labeling

Organoids were incubated with AM1-44 dye solution (10 μM, Biotium, 70038) in NB media for 30 seconds at room temperature. Under these conditions, the dye has been demonstrated to enter hair cells via mechanotransduction channels (Gale et al., 2001; Meyers et al., 2003; Herget et al., 2013). After incubation the sample was immediately fixed with 4% paraformaldehyde for 1 h on ice and incubated overnight at 4°C with 20% sucrose, embedded with O.C.T. compound and sectioned using a cryostat to obtain 12–14 μm sections. Nuclei were visualized using DAPI counterstain. Samples were mounted onto glass slides using moviol mountant followed by image capture using a ZEISS Observer z1 fluorescence microscope.

### Helium Ion Microscopy

Samples were washed using DPBS and fixed with 2.5% paraformaldehyde/2.5% glutaraldehyde for 1 hour on ice. This was followed by sequential dehydration with ethanol (30, 50, 75, 85, 95, and 100%: 15 min washing). Fixed samples were then dried by means of a critical point drier (Balzers CPD 030, BAL-TEC) with performing 8 exchange cycles of CO_2_. All additional fill, heating, and venting steps were performed at medium speed as well. After drying, the samples were carefully removed and adhered to double-sided copper tapes on aluminum stubs. Samples were imaged via the Helium Ion Microscope (HIM) (Carl Zeiss, Orion Nanofab) operating at an accelerating voltage of 30 and a beam current of ~0.5 pA. No further metallic coating was performed since the HIM is armed with a very low voltage electron gun (flood gun) to compensate positive surface charge accumulation on the insulating biological samples. Under these experimental conditions, no obvious beam damage or change in morphology was observed on the samples surface. During imaging the electron beam energy and the X and Y deflectors were adjusted correspondingly to ensure that the best possible image could be obtained.

### Kinocilia Length Measurement

To compare the length of longer stereocilia and kinocilia in RCCS-derived organoid and human fetal vestibular tissue, respectively, ImageJ software was used to estimate the measurements from images taken using HIM (Carl Zeiss, Orion Nanofab) by manually drawing straight lines along the cilia. Estimating the length of curved cilia was performed by drawing two straight lines which intersect at the inflection point of the cilium. An example is provided in Supplementary Figure 3. HIM pictures of the samples were taken at same magnification and inclination. The cilia length was documented in μm and the raw data of *n* = 6 measurements from *n* = 1 organoid and *n* = 1 fetal tissue are shown in Supplementary Figure 3. Cilia length data in **Figure 2** are shown as mean ± SD. For comparison of cilia length, statistical analysis was performed using GraphPad Prism 7 software. Data passed the Normality Test with Shapiro-Wilk method and alpha = 0.05, showing a normal distribution. Statistical analysis to compare the means of the cilia lengths from the organoid and fetal tissue was performed using the *T*-Test with Holm-Sidak method and alpha = 0.05.

### Micro-Computed Tomography

Samples were washed with distilled water (Life Technologies) and placed on parafilm suspended in a CT scan tube and scanned with microCT (μCT50, Scanco Medical AG, Bruttisellen, Switzerland) at an energy of 55 kVp, an intensity of 72 μA, 0.5 mm Al filter, and an integration time of 1,500 ms, with a voxel resolution of 0.8 μm. Parafilm was also used to secure the samples from above and avoid dehydration. Contours were drawn to isolate samples from tube and parafilm. Data were filtered using a 3D constrained Gaussian filter with finite filter support (1 voxel) and filter width (σ = 1.2). A threshold was applied at 27% of the maximum grayscale value to separate the calcium components from the organoid and background. A second threshold from 10 to 26.9% was applied to separate the organoid from the background. In order to remove insignificant particles, morphometric component labeling was used to find all calcium components larger than 200 voxels (~100 μm^3^), and a histogram of the number of components and size of these components was produced. From this the mean component volume, as well as the largest and smallest components in a measurement were found. Additionally, the combined volume of all components in a measurement was calculated. The high resolution enabled capture of very small components, however, the organoid mass was too large to capture in its entirety at the same resolution; i.e., the data becomes too large to use. For this reason, measurements of some samples were made in two stacks. As organoids have a very low x-ray attenuation and are therefore difficult to segment, to avoid artifact detection, components smaller than 200 voxels were eliminated. This counteracted the issue, but in turn could also eliminate viable components of interest.

### Electrophysiology

To perform recordings of inner ear organoids, vesicle-like structures were dissected, opened and flatted on a glass coverslip with the outer side facing up. A net was used to hold the organoid vesicle in place on the coverslip. The organoid vesicles are transferred to a recording chamber containing oxygenated Liebovitz's L15 cell culture medium (containing in mM; 1.26 CaCl_2_, 0.98 MgCl_2_, 0.81 MgSO_4_, 5.33 KCl, 0.44 KH_2_PO_4_, 137.93 NaCl, 1.34 Na_2_HPO_4_, 5 Na-pyruvate; Life Technologies, Australia; pH 7.45, 305 mOsM) and perfused at a rate of 2 bath volumes/min. Whole cell patch clamp recordings were done using borosilicate glass microelectrodes (3–5 MOhm; King Precision Glass Inc., CA, USA) filled with potassium gluconate internal recording solution containing (in mM); 42 KCl, 98 K.gluconate, 4 HEPES, 0.5 EGTA, 1 MgCl_2_, 5 Na.ATP. All experiments were done at room temperature (22°C). Recording from developing human fetal hair cells has been approved by The University of Newcastle Human Ethics Committee and was done as previously described (Lim et al., 2014). Briefly, inner ears were isolated from the products of conception aged 12–14 weeks gestation in an ice-cold modified glycerol artificial cerebrospinal fluid (ACSF) containing (in mM) 250 glycerol, 26 NaHCO_3_, 11 glucose, 2.5 KCl, 1.2 NaH_2_PO_4_, 1.2 MgCl_2_, and 2.5 CaCl_2_ bubbled with 5 % CO_2_/95% O_2_. The vestibular triad comprising the anterior and horizontal cristae ampullares and utricle were dissected and placed in the recording chamber. Recordings were made in Liebovitz'a L15 cell culture media, as described above for organoid vesicles. Cells were visualized using infrared differential interference contrast (IR-DIC) optics. Recordings were obtained using an Axopatch 200B amplifier running Axograph X software and sampled at 20 kHz and filtered at 2–10 kHz. Voltage protocols were used to characterize cell type. Instantaneous tail currents (at *t* = 0, when switched to −30 mV from membrane potential range −120 to +20 mV) were measured and used to plot activation and deactivation curves. These were fitted using the Boltzmann equation (Lim et al., 2011) to calculate G_max_, the maximum conductance; V_½_, potential at half-activation; and S, voltage required for an *e*-fold change in conductance. No correction was made for liquid junction potential (~–4 mV) and no leak subtraction was used. The Shapiro-Wilk test for normality (SPSS) showed the majority of electrophysiological data were normally distributed. Consequently, data were analyzed using independent sample *t*-tests. G_Max_ data for Na^+^ channels were not normally distributed and were analyzed using Mann U Whitney test. Data are described as mean values ± SD. “*n*” refers to the number of recorded cells.

### Representative Data and Reproducibility

Derivation of organoids was replicated 7 times in independent experiments from 2 hES cell lines, H3 and H9, and 1 hPS cell line, 007-5, with a total of 38 organoids assessed for ATOH1 expression. Efficiency of our protocol was assessed through a semi-quantitative analysis of ATOH1 expression in hPSC-derived organoids at different timepoints (from 7 DIV to 133 DIV) as number of ATOH1+ clusters where each cluster counts >8–10 cells. Immunofluorescence images for hair cell markers, MYO7A and CTBP2, are representative of at least *n* = 3 organoids derived from *n* > 3 independent experiments.

## Results

### Generation of Neural Organoids From hPSCs Using Microgravity

**Figure 1:**
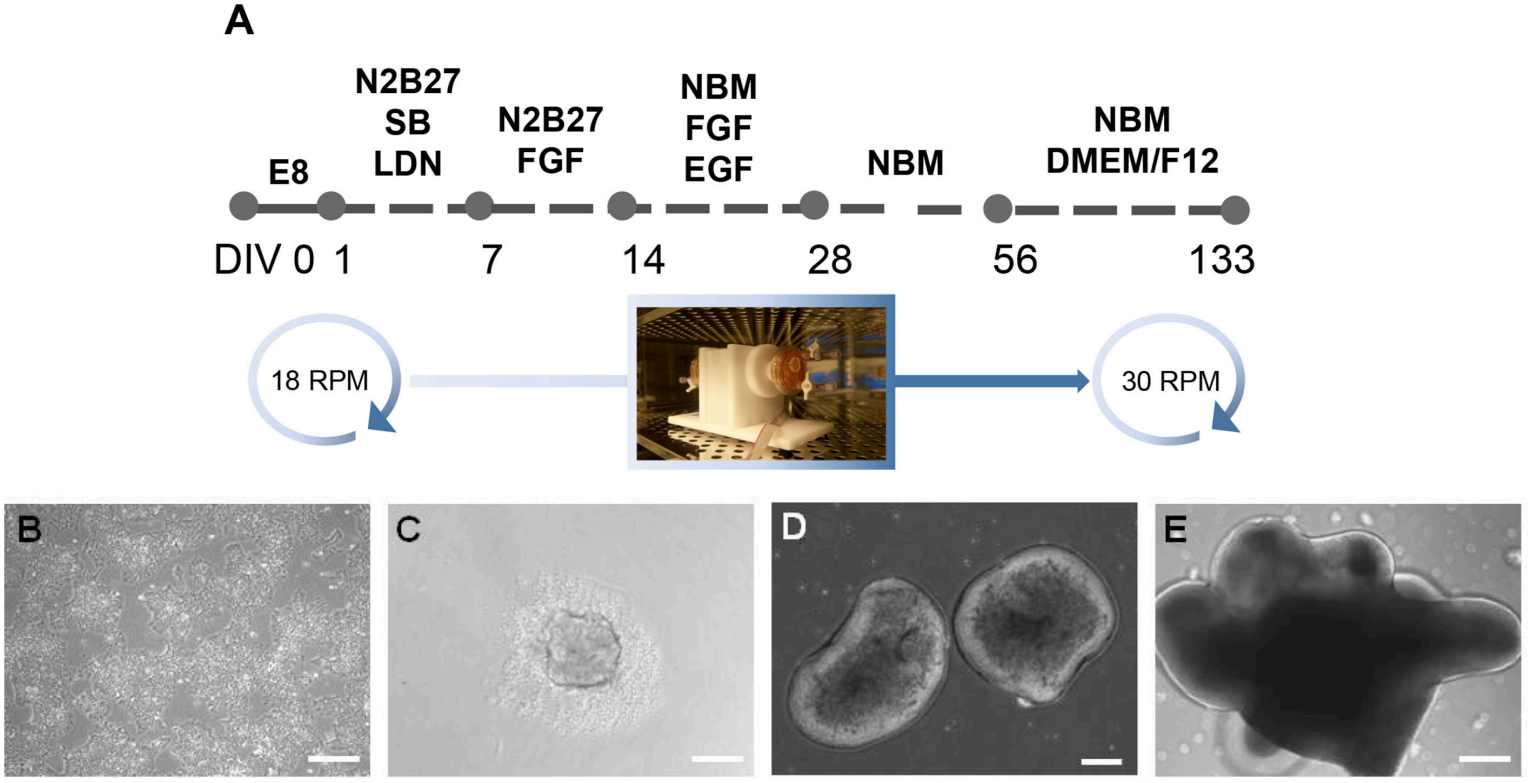
Generation of inner ear organoids using the RCCS. **(A)** Schematic overview of RCCS protocol where an initial and final speed rotation of 18 and 30 RPM (rotations per minute) respectively, was applied. Photo of RCC set up in the tissue culture incubator was taken by Stefano Frausin (University of Melbourne). **(B)** HPSCs maintained as bulk culture at 0 DIV. **(C)** HPSCs-derived aggregate at DIV 1. HPSCs-derived organoids at **(D)** 14 and **(E)** 56 DIV. Scale bars, **(B,D,E)** 200 μm, **(C)** 100 μm.

Our former study described a dynamic three dimensional (3D) RCCS to support derivation of neural organoids from hPSCs (Mattei et al., 2018). The RCCS was initially utilized at the National Aeronautics and Space Administration (NASA) to culture 3D cellular aggregates under microgravity in order to investigate the biological effects of such conditions on human tissues (Wolf and Schwarz, 1991). The RCCS offers advantages over other static organoid culture systems because it sustains long-term cultures by providing a continuous fluid flow that enables efficient transfer of oxygen and nutrients together with exchange of waste (Carpenedo et al., 2007). We previously reported that hPSC-derived neural organoids generated in the RCCS were biased to midbrain-hindbrain fate, as shown by upregulated expression of *ENGRAILED1, HOXA2*, and *GBX2* during neural induction (Mattei et al., 2018). Furthermore, as early as 14 days (14 DIV) within the RCCS, organoids develop as irregular shapes that later form numerous vesicular protrusions with a bright appearance surrounding an inner dense core (Figure 1). The described vesicular morphology, combined with previous observations reported by Hashino et al. (Liu et al., 2016; Koehler et al., 2017) and the dorsal specification of our hindbrain-committed organoids suggested by expression of PAX7 (Supplementary Figure 1A) encouraged us to further investigate the presence of dorsal hindbrain-derived structures such the inner ear tissue.

### Organoids Are Enriched With Hair Cell-Like Cells on Their Outer Surface

**Figure 2:**
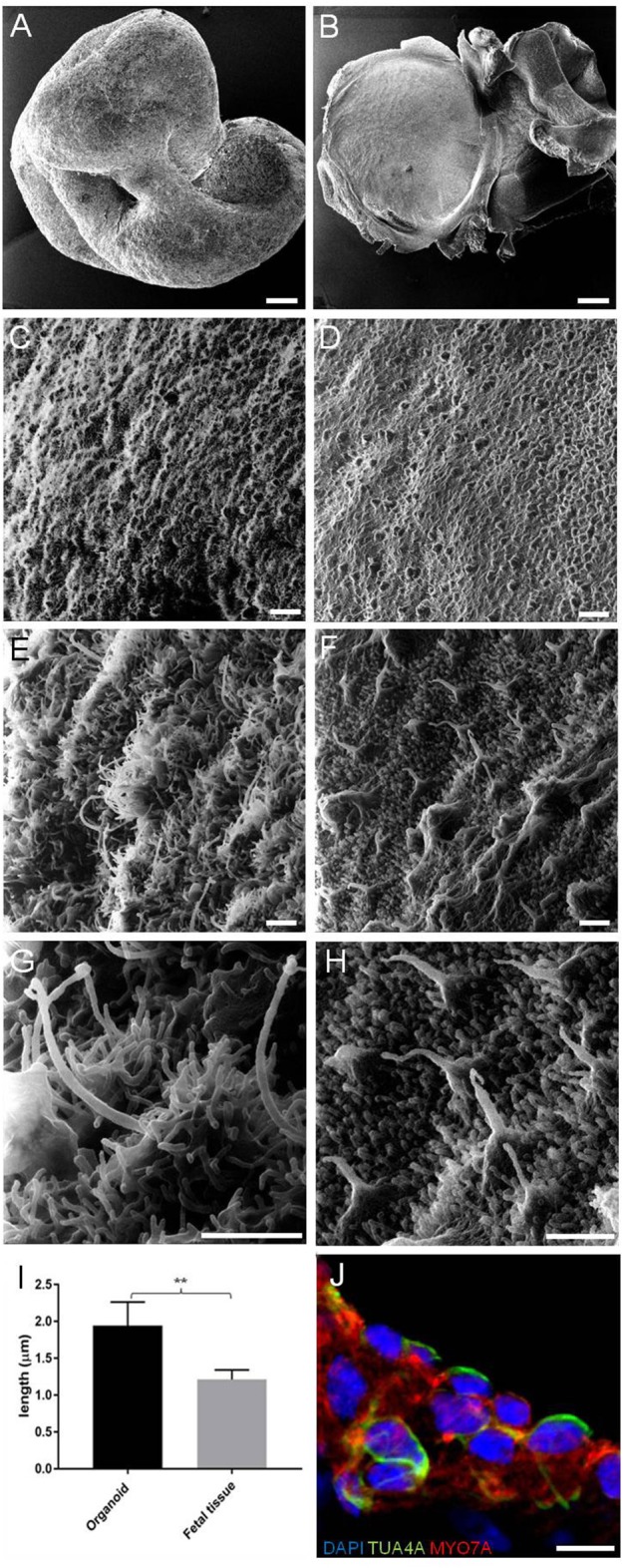
HIM images of RCCS-derived organoids and human fetal utricle tissue. **(A)** Low magnification imaging of whole hESCs-derived organoid at 56 DIV. **(B)** Human fetal utricle at 10 weeks (~day 70) of gestation. **(C,D)** Ciliated epithelium in organoid **(C)** and in macula of fetal utricle **(D)**. **(E–H)** High magnification images showing longer cilia on organoid's surface **(E,G)** resembling kinocilia in fetal utricle **(F,H)**. **(I)** Comparison of the mean (± SD) length of longer cilia in organoid and kinocilia in fetal tissue (*n* = 6 measurements each from one organoid and one fetal tissue) (^**^p < 0.001). *T*-Test was used to assess significance. **(J)** Hair cells in organoid expressing TUBA4A and MYO7A. Scale bars, **(A,B)** 100 μm, **(C,D)** 5 μm, **(E,F,G,H)** 1 μm, **(J)** 10 μm.

We first investigated the ultrastructural morphology of our inner ear organoids at 56 days *in vitro* (DIV) using helium ion microscope (HIM; Figure 2). The HIM analyses revealed that the surface of the organoid consisted of a dense layer of ciliated cells (Figure 2C) protruding homogeneous bundles with occasional single longer cilia (Figures 2E,G). The surface morphology of the organoids closely resembled aspects of the human fetal vestibular apparatus aged 10 weeks gestation (~70 days; Figures 2B,D,F,H), which corresponds to when vestibular hair cells express hair cell specific marker, myosin VIIa. However, cochlear hair cells don't express myosin VIIa until week 12 of gestation in human (Locher et al., 2013; Lim and Brichta, 2016). At high magnification, we observed single elongated cilia on surface of the organoid, similar in morphology to the kinocilia of human fetal utricular hair cells (Figures 2G,H,I). The kinocilium-like phenotype found within organoids was further supported by expression of alpha-acetylated tubulin (TUBA4A) in single, hair-like protrusions on the apical surface of the hair cells (Figure 2L; Lim and Brichta, 2016; Koehler et al., 2017).

Taken together, these data suggest hPSC-derived organoids are comprised of large populations of ciliated cells, consistent with the developing human inner ear.

### Development of Inner Ear Accessory Structures Within Organoids

**Figure 3:**
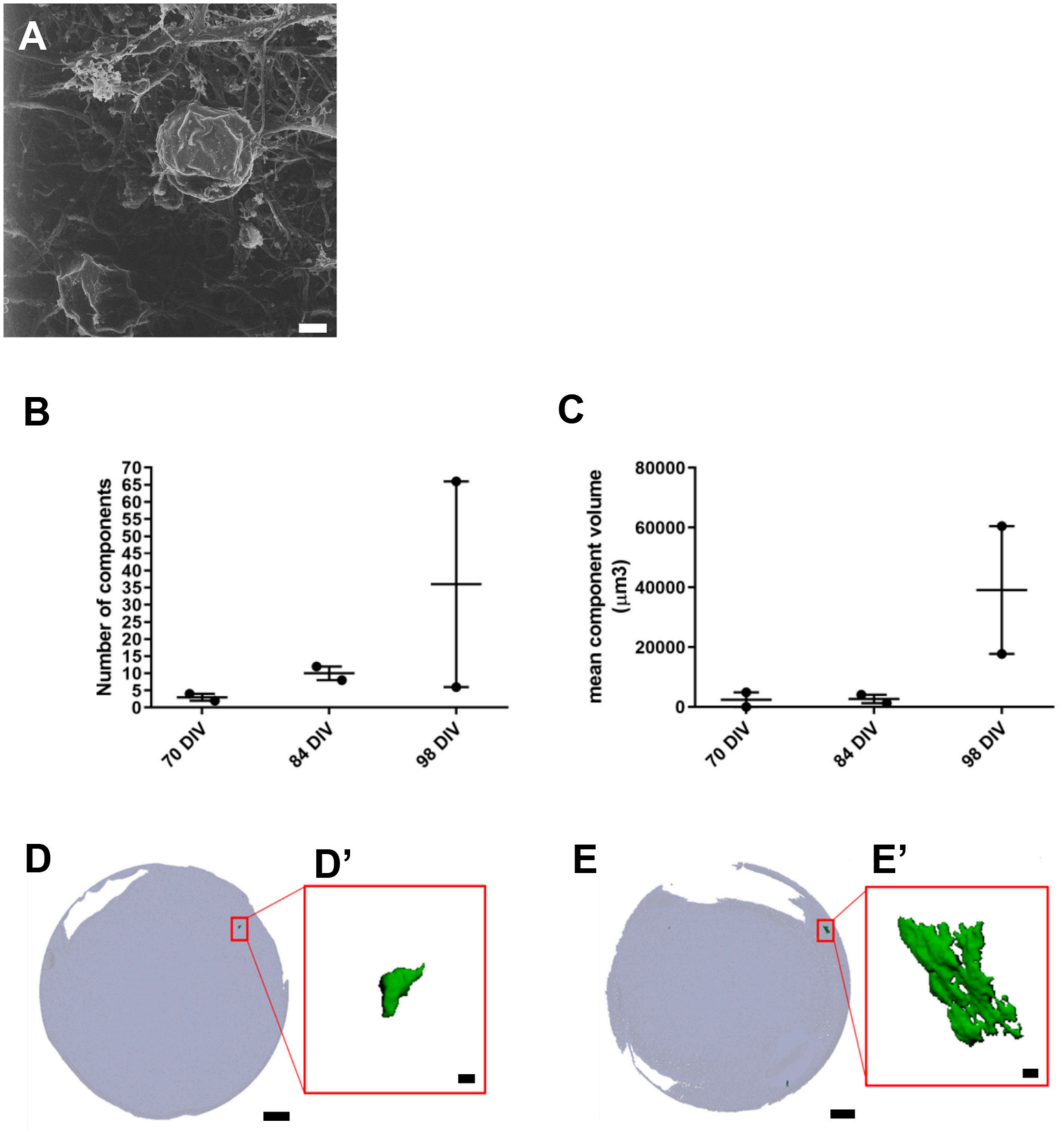
Presence of inner ear crystalline structures in RCCS-derived organoids. **(A)** HIM image of organoid at 98 DIV shows the presence of a filamentous membrane covering the surface of organoid (arrow and otoconia-like structures (arrowhead). **(B–E)** Micro-computed tomography of organoids reveals the presence of mineral components suggestive of otoconia. Total component volume **(B)** and mean component volume **(C)** of organoids at 70, 84, and 98 DIV (*n* = 2 at each timepoint). Data shown as mean with range and individual values plotted. Representative micro-CT images of whole organoid scan at 70 DIV **(D)** and 98 DIV **(E)** and magnified three-dimensional reconstructions **(D',E')** of one mineral component. Scale bars, **(A)** 2 μm, **(D,E)** 200 μm, **(D',E')** 10 μm.

We also used HIM to assess morphological changes in organoids cultured for longer periods. High magnification imaging revealed that the surface of mature organoids at 98 DIV consist of some crystalline-like structures partially embedded in a loose filamentous matrix which resemble developing otoconial membrane and otoliths (Figure 3A). To further examine whether the structures were crystalline in their molecular structure, we employed micro-computed tomography (CT) of whole inner ear organoids at 70, 84, and 98 DIV time points. At all three timepoints, scans revealed mineral components within the organoids which increased in number (Figure 3B) and volume (Figure 3C) with time in culture. Mineral components were primarily located on the surface of the organoid surface, with a smaller number located internally (Figures 3D,E) (Supplementary Table 2). The three-dimensional reconstruction of imaged structures revealed irregular and crystalline-like shapes (Figures 3D',E') that may resemble the typical barrel-shaped morphology of human otoconia (Sánchez-Fernández and Rivera-Pomar, 1984).

Overall these data provide novel evidence of inner ear vestibular-like phenotype of the hPSC-derived organoids with the presence of calcium carbonate otoconia, which are a distinguishing feature of vestibular system. Significantly, the presence of otoconia-like structures within the hPSC-derived organoids also demonstrates their capacity to form complex, multilayered structures analogous to human inner ear tissue.

### RCCS-Derived Organoids Express Hair Cell Specific Markers Consistent With Inner Ear Development

**Figure 4:**
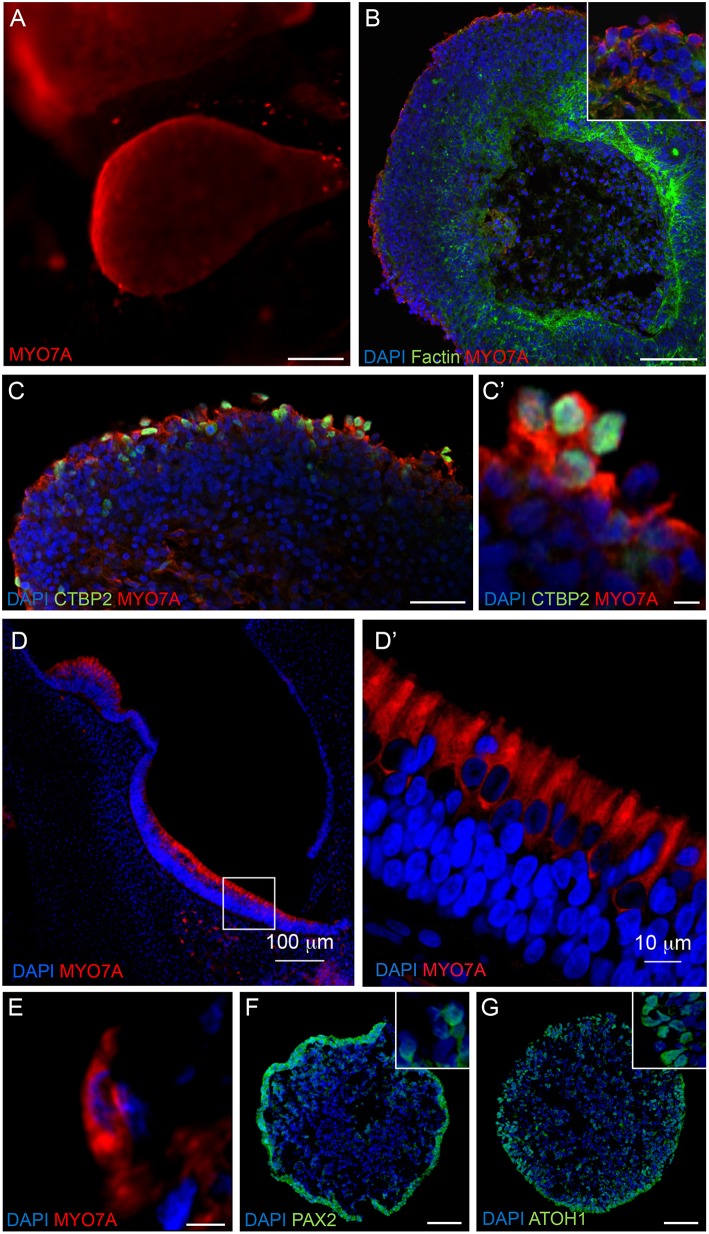
Expression of hair cell and otic placode markers in RCCS-derived organoids. **(A)** HESC-derived organoids show MYO7A+ protrusions on surface at 56 DIV. **(B,C)** cells in the outer epithelium of vesicles expressing MYO7A and f-ACTIN **(B)** as well as CTBP2 **(C,C')** at 35 DIV. **(D–E)** Human fetal saccule at 11 WG consists of hair cells expressing MYO7A **(D)** which defines the typical cylindrical shape of type II hair cells **(D')** and similarly detected within organoid at 35 DIV **(E)**. **(F,G)** Expression of PAX2 **(F)** and ATOH1 **(G)** in occasional otic-pit-like clusters of cells in the outer epithelium of hiPSC-derived organoid at 7 DIV. Scale bars, **(A)** 200 μm, **(B,D)** 100 μm, **(C,F,G)** 50 μm, **(D')** 10 μm, **(C',E)** 5 μm.

Immunostaining analyses were performed on RCCS-derived organoids to examine expression of hair cell markers that are found in the developing human inner ear. High expression of myosin VIIa (MYO7A), a hair-cell specific marker, was observed within the organoid's cystic-like protrusions at 56 DIV (Figures 4A,B). Images of sectioned organoids at 35 DIV show an enriched population of cells positive for MYO7A and the hair cell ribbon synapse specific marker, CTBP2 (MYO7A+/CTBP2+) on the outer surface (Figure 4C). The expression of these hair cell markers on the surface of the organoid is consistent with results from HIM analyses. Interestingly, CTBP2 expression was predominantly localized to the nucleus (Figure 4C'). At a high magnification we observed several MYO7A+ cells showing a typical hair cell-like shape (Figure 4E) comparable with MYO7A+ hair cells in the human fetal vestibular system (Figures 4D,D').

Immunostaining analyses of PAX2 and ATOH1 were also performed at earlier stages of organoid formation, which are key factors involved in otic placode formation and inner ear hair cell specification, respectively. Immunostaining of 7 DIV organoids show expression of PAX2 and ATOH1 particularly in the outer cellular layers (Figures 4F,G). ATOH1 expression persists long-term as it was also detected in organoids cultured up to 133 DIV (Supplementary Table 1).

Cells expressing PAX6, a neural progenitor marker, were also found within the organoids at 21 DIV (Supplementary Figure 1B), however, these cells were mainly localized within the internal regions of the organoids and separate to the PAX2+ pool (Figure 4F). Cells positive for SOX2 expression, a marker of supporting cells that surround inner ear hair cells and also in type II vestibular hair cells (Oesterle et al., 2008), were also observed in the organoids at 35 DIV (Supplementary Figure 1C). Consistent with expression of neural markers within younger organoids, high levels of βIII Tubulin expression was observed within the organoids at 49 DIV (Supplementary Figure 1D), which appeared to penetrate into the outer MYO7A+ sensory epithelial layer (Supplementary Figure 1D').

Taken together, RCCS-derived organoids show expression of inner ear progenitor, hair cell, and neuronal markers at different time points of differentiation, suggesting that the growing organoids recapitulate aspects of inner ear development.

### Functional Assessments of Organoid-Derived Hair Cells and Comparison to Human Fetal-Derived Vestibular Hair Cells

**Figure 5:**
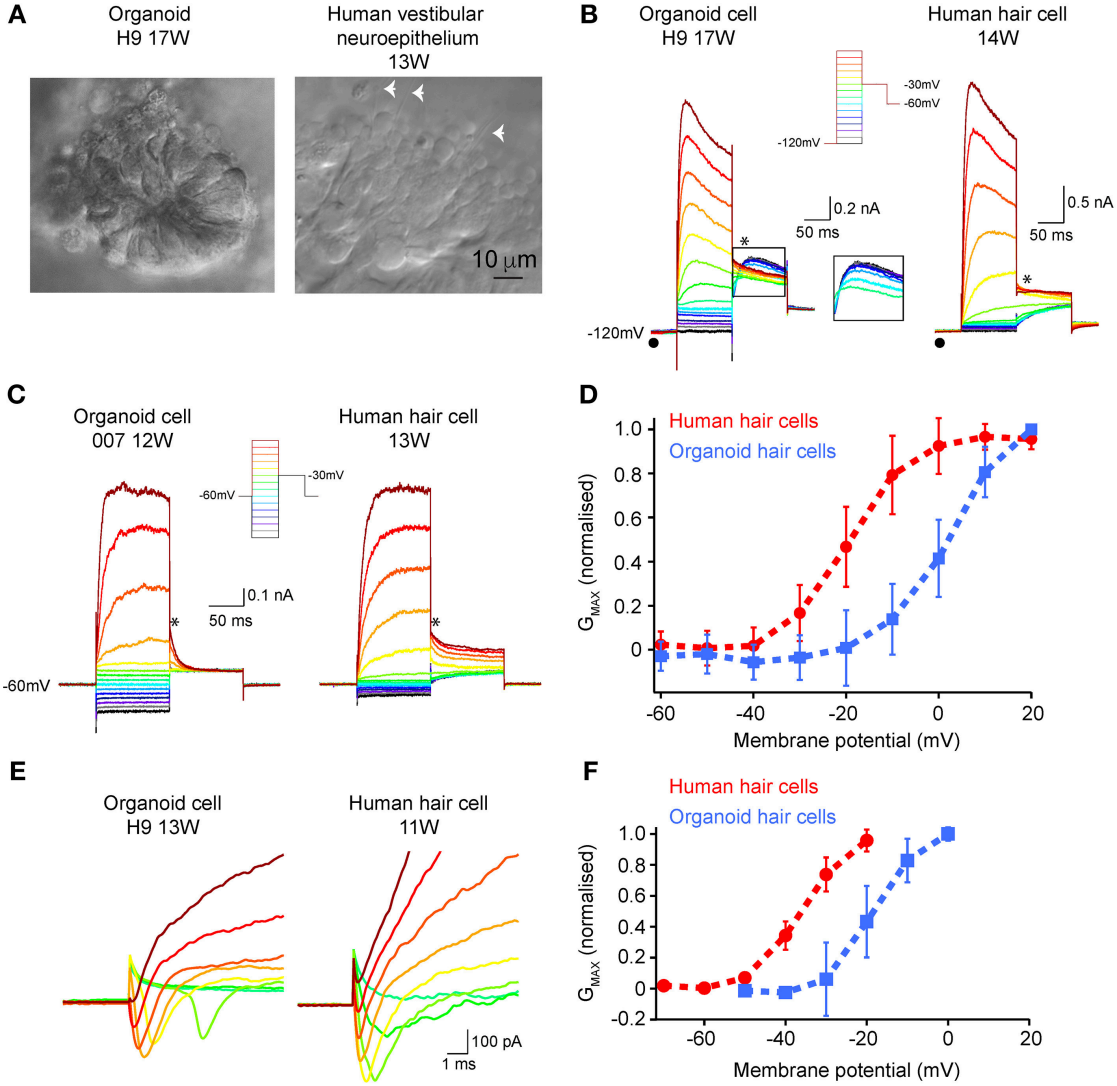
Electrophysiological characterization of hair-cell like organoid cells and human fetal vestibular hair cells. **(A)** Infra-red differential interference contrast optics images of organoids and human vestibular epithelium aged 17 weeks in culture and 13 weeks gestation, respectively. The white arrowheads in right panel show hair bundles. **(B)** Using a hyperpolarizing pre-pulse to −120 mV (inset), organoid cells (left) and human vestibular hair cells (right) show fast activating outward currents, presumably K^+^ currents which vary in amplitude. The pre-pulse to −120 mV (λ) does not elicit the type I hair cell specific G_k,l_ current in any organoid or human fetal hair cells. In some organoid cells there is evidence for an “A-like” current when analyzing tail currents (^*^), that is not observed in developing human hair cells. **(C)** Using a type-II hair cell voltage protocol (inset), organoid cells, and human hair cells have similar characteristics, with outward currents that are inactivating. Tail currents were measured (^*^) and used to calculate Gmax, V_1/2_, and slope. **(D)** Normalized conductance– voltage plots of tail currents recorded in organoid cells (*n* = 6 cells; blue line) and human fetal hair cells (*n* = 10 cells; red line) show differences in V_1/2_. Data are mean ± SD **(E)** Inward presumptive Na^+^ currents in organoid (left) and human vestibular hair cells (right) aged 11 weeks and 13 weeks respectively. **(F)** Normalized conductance—voltage plots of inward Na^+^ currents show more depolarized V_1/2_ in organoid cells (*n* = 9 cells; blue line) compared to human fetal hair cells (*n* = 10 cells; red line), while slopes are similar. Data are mean ± SD.

Patch clamp analyses were conducted to assess functional properties of organoid-derived hair cell-like cells in comparison with human fetal hair cells. We recorded from a total of 27 cells from organoids arising from three independent hPSC lines (H9, H3, and 007). In some organoids, infra-red differential interference contrast optics showed the presence of neuroepithelial-like rosettes (Figure 5A, left), which contained hair cell-like cells. Human fetal vestibular hair cells are shown in Figure 5A (right). We are not able to definitively determine vestibular hair cell type by morphology in human vestibular neuroepithelium at any stage of development examined (10–16 WG). However, using a voltage-activated protocol (Figure 5B, inset) we are able to determine whether a hair cell has type I, or type II vestibular hair cell characteristics, or non-hair cell characteristics. Of the 27 recorded cells from organoids, 15 had whole cell conductances consistent with those from mammalian type II vestibular hair cells. Our analysis focused on these 15 organoid cells with type II vestibular hair cell like characteristics. In our recordings from organoid cells (aged between 10 and 17 weeks in culture) there was no evidence of any cells possessing the type I hair cell specific G_K,L_ conductance (•). Neither the organoid cell aged 17 weeks in culture or vestibular hair cell aged 14 weeks gestation showed type I hair cell characteristics (Figure 5B). Using the protocol with hyperpolarizing prepulse (−120 mV) and a ladder of voltages from −120 to +20 mV (inset), we observe in both organoid cells and developing human hair cells, fast outward currents that are consistent with K^+^ currents. Of the 15 organoid cells, 20% had outward K^+^ currents that inactivated by 10% from peak maximum amplitude to steady state amplitude at +20 mV (Figure 5B, left). Tail currents (^*^) also showed the presence of an A-like current (outlined inset), in a subset of organoid cells with otherwise type II hair cell characteristics. Using the type II hair cell voltage-activation protocol (Figure 5C, inset), a series of depolarizing steps from −120 to +20 mV shows outward currents, consistent with K^+^ currents in both organoid cells and developing human vestibular hair cells. There is variation in the maximum peak current amplitude at +20 mV between organoid cells and developing human hair cells. Analysis of tail currents after stepping from a test voltage (−120 to +20 mV) to −30 mV in organoid cells and developing hair cells showed significant differences in G_MAX_ between organoid cells (2.25 ± 0.28 nS, *n* = 6) and hair cells (5.55 ± 0.08 nS, *n* = 10, *p* < 0.05) (Figure 5D; Supplementary Table 3). Normalized fits of the conductance—voltage data also showed that organoid cells had more depolarized V_½_ compared to developing human vestibular hair cells (organoid cells: 2.60 ± 0.62 mV, *n* = 6 vs. human hair cells:−19.58 ± 0.38 mV, *n* = 10, *p* < 0.05) but similar slopes (organoid cells: 7.10 ± 0.47 nS.mV, *n* = 6, human hair cells: 6.24 ± 0.35 nS.mV, *n* = 10, *p* > 0.05). A total of 9 (of 15) organoid cells showed the presence of fast activating, fast inactivating inward currents that are consistent with Na^+^ currents (Figure 5E, left) that are also present in developing rodent (Wooltorton et al., 2007) and human vestibular hair cells (Figure 5E, right; Lim et al., 2014). The conductance of presumptive Na^+^ channels in organoid cells was higher than those in human vestibular hair cells but were not statistically significantly different (G_MAX_ = 10.93 ± 0.45 nS, *n* = 9 vs. 5.82 ± 0.17, *n* = 10, respectively, U = 33, *p* = 0.327). Normalized fits of the presumptive Na^+^ conductance—voltage plots showed organoid cells were significantly more depolarized V_½_ than human hair cells (organoid cells: −18.45 ± 0.64 mV, *n* = 9, human hair cells −36.5 ± 0.62 mV, *n* = 10, *p* < 0.05) but similar slopes (organoid cells; 5.36 ± 0.60 nS.mV, *n* = 9 vs. human hair cells 5.32 ± 0.54 nS.mV, *n* = 10, *p* >0.05) (Figure 5F; Supplementary Table 3). These electrophysiological results show voltage-activated currents between human derived hPSCs organoids and human fetal vestibular hair cells are similar.

Active mechanotransduction channels may be detected by examining cellular uptake of styryl dye AM1-44 (Gale et al., 2001; Meyers et al., 2003; Herget et al., 2013). Brief application of AM1-44 to organoids (84 DIV) showed fluorescence was present in distinct and discrete cellular regions at the organoids' surface (Supplementary Figure 2). These results suggest active mechanotransduction channels may be present in organoids, however this needs to be validated with additional studies such as recording currents elicited from controlled mechanical stimulation.

## Discussion

Investigators worldwide recognize the value of hPSCs and more recently, the dynamic 3D culture systems, as unique means for *in vitro* modeling. While overcoming technical limitations is an ongoing challenge, important findings for developmental studies and *in vitro* disease modeling have been achieved for several tissues of interest, including neural tissue (Kadoshima et al., 2013; Lancaster et al., 2013; Mariani et al., 2015; Muguruma et al., 2015; Qian et al., 2016; Birey et al., 2017; Kuwahara et al., 2017). This includes generation of inner ear organoids from mouse and human PSC first reported by Hashino and colleagues (Liu et al., 2016; Koehler et al., 2017). These studies used an ATOH-1 reporter cell line to monitor hair cell development in mouse and subsequently in human PSC derived-organoids alongside immunohistochemical and electrophysiological analyses. Here, we demonstrate that inner ear organoids can also be generated from hPSC using RCCS, however with some significant advancements. Using HIM, micro-computed tomography, and electrophysiology we show unique structural, functional, and phenotypic properties of immature vestibular hair cells and associated otoconia that closely resemble the human fetal vestibular system. To the best of our knowledge, this is the first study to directly compare structural and functional properties of hPSC-derived inner ear organoids with human fetal vestibular tissue. Significantly, we also describe the formation of otoconia, thereby demonstrating a more structurally complete model of inner ear development.

The formation and maturation of RCCS hPSC-derived organoids mimic distinct stages of human inner ear development with a similar temporal profile. The human inner ear first arises from a patch of otic placode progenitors surrounding the rhombomere 5 of dorsal hindbrain (Bruska et al., 2009). Similarly, a layer of cells expressing PAX2, an otic placode marker, were identified on the surface of organoids during the first week of neural induction. By 3 weeks in culture, vesicle protrusions were forming from the organoid, which may mimic the otic-like vesicles observed in human fetal inner ear (Koehler et al., 2017). Expression of ATOH1 is crucial for all stages of inner ear development, thereby being a major marker for tracking hair cell induction and maturation (Bermingham et al., 1999; Shailam et al., 1999). Using immunofluorescence analyses, ATOH1 expression was observed within the hPSC-derived organoids at several time points in culture ranging from 7 to 133 DIV. During neural induction stages, ATOH1 shows a similar spatial expression to PAX2, which may be associated with induction of otic placode like-progenitors. Of note, ATOH1 expression is detected in at least 58% of organoids, demonstrating the efficiency and robustness of the RCCS protocol for deriving hindbrain-like organoids that supports inner ear specification. Maturation of hPSC-derived hair cells within the organoid vesicles followed an inside-out radial differentiation pattern, whereby MYO7A+ cells were mainly found on the outer edge of vesicles and βIII tubulin-expressing neurons within the vesicle/organoid center. This pattern is contrary to *in vivo* inner ear development and may be caused by the physical forces of fluid flow imposed on the organoids generated by a rotating system, which may affect tissue polarization as previously described (Chen et al., 2000; Helmke and Davies, 2002; Helmke, 2005; Mammoto and Ingber, 2010). The neuronal-epithelial regions found in the organoids is consistent with inner ear development whereby the vestibular nerve begins to innervate the undifferentiated sensory epithelium by 7 WG (Lim and Brichta, 2016). The neuronal component may arise from the PAX6+ pool detected within the younger organoids, further supporting a model of inner ear neurogenesis.

HIM showed a dense layer of ciliated hair-like cells spanning across the surface of the organoid at 56 DIV. The images clearly showed the presence of kinocilia, a unique feature of vestibular hair cells on the organoid surface that closely resembled the morphology of human fetal vestibular tissue at ~70 days gestation. It should be noted that during development, auditory hair cells also express a long stereocilia, resembling a kinocilium, however these are lost during development beginning at 24 weeks gestation (Igarashi, 1980; Lavigne-Rebillard and Pujol, 1986) and are not found in mature adult human cochlea. The maturation of hair cell-like cells was shown by functional MET channels and voltage-activated currents. While stereocilia have been described in electron microscopy studies of inner ear, few have described vestibular hair cell development of tip links where MET channels are localized. However, tip links and therefore presumably MET channels, are present by 14 WG in cochlea hair cells (Igarashi, 1980; Rhys Evans et al., 1985), which is consistent with AM1-44 results from organoids.

Electrophysiological analyses from organoid-derived cells shows these cells are electrically active and express a number of different ion channels. Organoid cells showed the presence of voltage-gated Na^+^ and K^+^ channels in hair cells. Typically, voltage activated K^+^ conductances in organoid like cells resemble those recorded from human fetal type II hair cells (Lim et al., 2014). The conductance of K^+^ channels in fetal hair cells was greater than organoid cells suggesting a higher proportion of functional K^+^ channels in fetal hair cells at the ages examined. Interestingly, however there was also a subset of organoid cells that also have a fast activating and fast inactivating outward K^+^ current that are consistent with “A-like” currents that are not observed in fetal human hair cells. A-like currents however, have been recorded from isolated adult human vestibular hair cells (Oghalai et al., 1998). Like human fetal hair cells (Lim et al., 2014) and developing rat hair cells (Wooltorton et al., 2007), there were also a subset of organoid cells that expressed Na^+^ conductances. However, the kinetics of Na^+^ channel activation was more depolarized in organoid cells than human fetal or developing rodent vestibular hair cells. These electrophysiological results from organoid cells supported anatomical findings which showed immunofluorescent labeling of hair cell specific markers and MET channel results. Importantly, comparing the results from organoid cells to human fetal vestibular hair cells shows organoids derived from RCCS are functional and express a heterogeneity of hair cell types.

Following maturation of hair cell-like cells, using micro-computed tomography, we were able to detect increasing amounts of mineral deposits within the growing organoids that are consistent with formation of inner ear otoconia which are detected in human fetal tissue as early as 7 WG with the otoconial membrane being fully mature by 22 WG (Lim and Brichta, 2016). Loss and dislodgement of otoconia have been associated with benign paroxysmal positional vertigo, age-related dizziness, and in response to trauma, particularly blast induced trauma (Ross et al., 1976; Lim, 1984; Thalmann et al., 2001; Jang et al., 2006; Zalewski, 2015). The capacity of the RCC hPSC-derived organoids to promote the development of otoconia may serve as a pioneering approach to model vestibular pathologies that involve otoconia.

Generation of organoids showing an inner-ear phenotype was performed under microgravity conditions, despite the organoids being fated toward a cortical phenotype using dual SMAD inhibitors. We and others have previously showed that microgravity may influence stem cell differentiation and signaling (Chiang et al., 2012; Lei et al., 2014; Mattei et al., 2018). It is not well understood how microgravity may influence cell fate and differentiation, however activation of Wnt signaling has been suggested to be involved (Lei et al., 2014) which is required for hindbrain development. Given the efficiency of ATOH1 expression, our findings suggest that RCCS may be a highly suitable platform for deriving cells of hindbrain lineages, such as the inner ear. Further studies are needed to determine how microgravity may enable stem cell/progenitor differentiation toward a hindbrain phenotype, which also provides insight into understanding inner ear development.

In summary, these data demonstrate efficient generation of organoids from hPSC using microgravity, which recapitulate some key structural and developmental components of human inner ear development, including the formation of functional vestibular-like hair cells, neurons, and otoconia. These findings support the RCCS system to be a valuable platform for further advancing complex *in vitro* models of the human vestibular system.

## Data Availability

All datasets generated for this study are included in the manuscript and/ or the supplementary files.

## Ethics Statement

This study was carried out in accordance with the recommendations of Australian National Health and Medical Research guidelines with written informed consent from all subjects. All subjects gave written informed consent in accordance with the Declaration of Helsinki. The protocol was approved by University of Melbourne Human Ethics committee (#1545384 and 1545394) for use of human pluripotent stem cell lines and recordings from developing human fetal hair cells has been approved by The University of Newcastle Human Ethics Committee (#H-693-0608 and H-569-0503).

## Author Contributions

CM: conception and design, collection and assembly of data, data analysis and interpretation, manuscript writing, final approval of manuscript. RL, GD, KS, and BAN: conception and design, financial support, provision of study material, collection, and assembly of data, data analysis and interpretation, manuscript writing, final approval of manuscript. HD and ZL: collection and assembly of data, data analysis and interpretation, final approval of manuscript. BN: provision of study material, collection and assembly of data, data analysis and interpretation, final approval of manuscript. MT: administrative support, provision of study material, data analysis and interpretation, final approval of manuscript. MD: conception and design, financial support, administrative support, provision of study material, collection and assembly of data, data analysis and interpretation, manuscript writing, final approval of manuscript.

### Conflict of Interest Statement

The authors declare that the research was conducted in the absence of any commercial or financial relationships that could be construed as a potential conflict of interest.

## Supplementary Material

Supplementary Figure 1 Expression profile of RCCS-derived organoids**. (A–C)** HESC-derived organoids show expression of dorsal hindbrain marker PAX7 **(A)** and neural marker PAX6 **(B)** at 21 DIV as well as supporting hair cell marker SOX2 **(C)** at 35 DIV, where white dash line delineates organoid boundary. **(D)** TUBβIII neural mass in hESC-derived organoid at 49 DIV with some processes spreading up to the outer surface and **(D')** infiltrating a MYO7A+ sensory epithelium. Scale bars, **(A,C)** 20 μm, **(B,D)** 50 μm, **(C,D')** 10 μm.

Supplementary Figure 2 Mechanotransduction of RCCS-derived organoids. HESC-derived organoid at 84 DIV is capable of uptake AM1-44 dye. Scale bar, 10 μm.

Supplementary Figure 3 Kinocilia length measurements. An example of procedure used for estimating kinocilium length in **(A)** organoid and **(B)** fetal tissue. Straight lines were drawn along the cilia (dash lines in red) to estimate the length. **(C)** Raw data of *n* = 6 kinocilium measurements each for an organoid and fetal tissue as plotted in Figure 2I. Scale bar, 1 μm.

Supplementary Table 1 Expression efficiency of ATOH1 in inner ear organoids. ATOH1 expression in organoids derived from hPSC lines (H3 hESC, H9 hESC and 007–5 iPSC lines) in n = 7 biological replicate experiments from 7 to 133 DIV. 58% of organoids showed ATOH1 expression.

Supplementary Table 2 Volume of components in each measurement by micro-computed tomography. The table shows the total number of components, as well as the mean, smallest and largest component volumes in each measurement, and the combined total volume of all components in a measurement. The two measurements (scans) of any single sample have not been pooled.

Supplementary Table 3 G_Max_, V_½_, and slope values of IV relationship of K^+^ and Na^+^ currents in organoid and human hair cells. Unless otherwise specified, all statistical analyses were independent sample *t*-tests. ^*^*p* < 0.05, + Mann U Whitney statistical analysis.
