# Supplementary figures and images for: Generation of Vestibular Tissue-Like Organoids From Human Pluripotent Stem Cells Using the Rotary Cell Culture System
Source: Front Cell Dev Biol. 2019 Mar 5;7:25. doi: 10.3389/fcell.2019.00025 (PMC6413170; doi:10.3389/fcell.2019.00025)

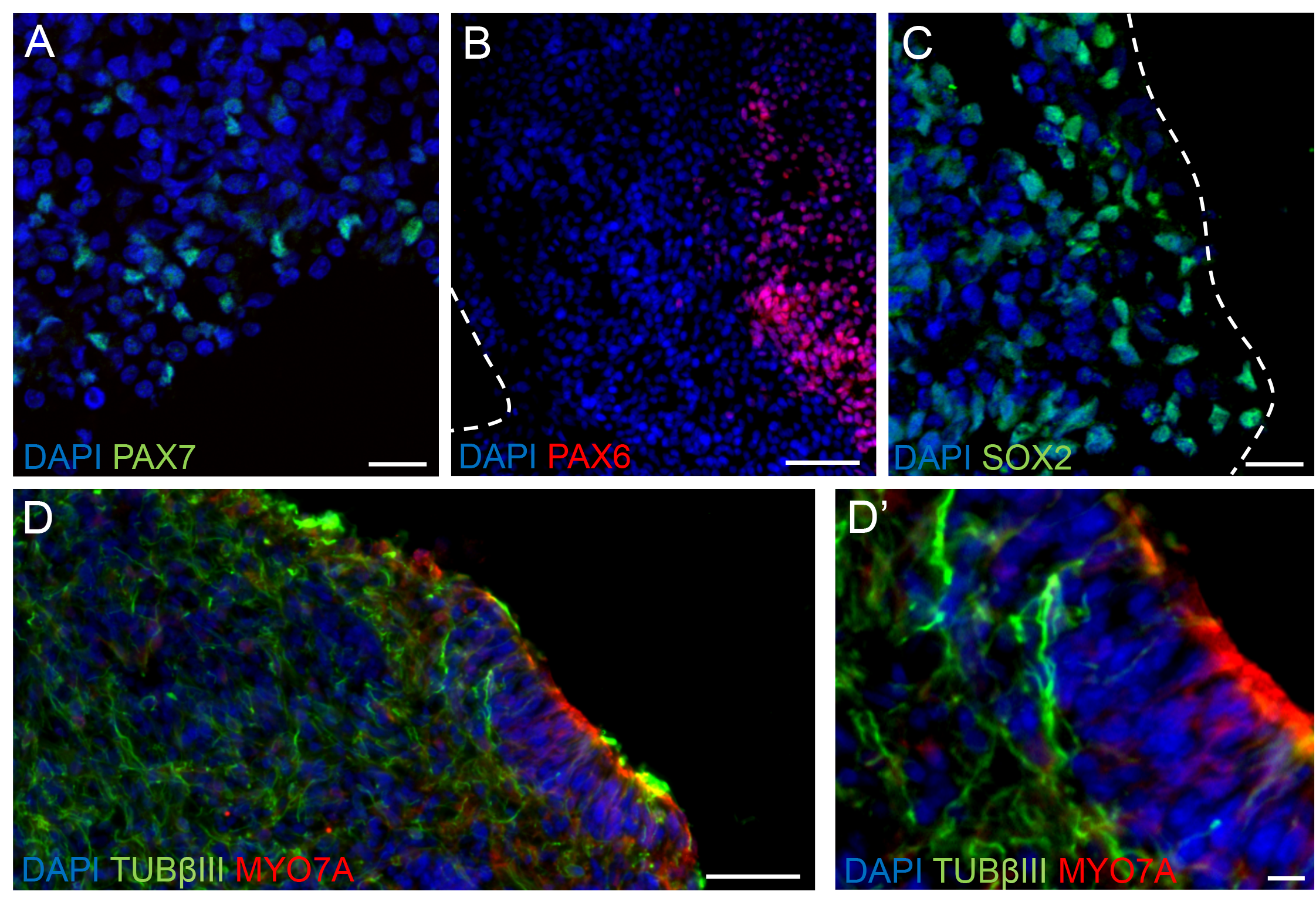

Supplement: Supplementary Figure 1 — Expression profile of RCCS-derived organoids. (A–C) HESC-derived organoids show expression of dorsal hindbrain marker PAX7 (A) and neural marker PAX6 (B) at 21 DIV as well as supporting hair cell marker SOX2 (C) at 35 DIV, where white dash line delineates organoid boundary. (D) TUBβIII neural mass in hESC-derived organoid at 49 DIV with some processes spreading up to the outer surface and (D') infiltrating a MYO7A+ sensory epithelium. Scale bars, (A,C) 20 μm, (B,D) 50 μm, (C,D') 10 μm. [file Image_1.TIF]

## Supplementary Figure 2

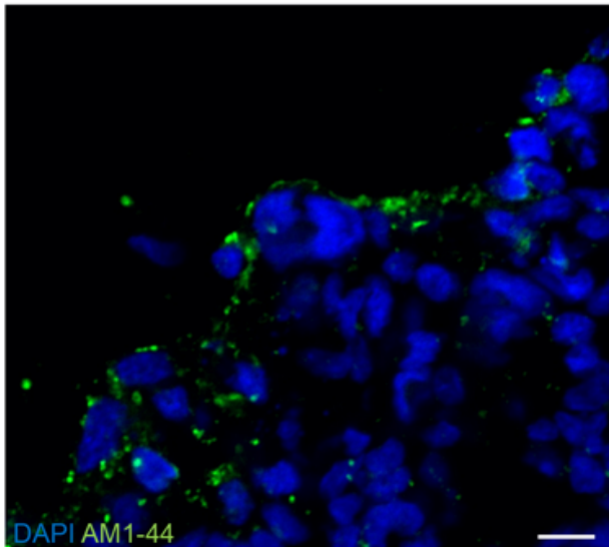

Supplement: Supplementary Figure 2 — Mechanotransduction of RCCS-derived organoids. HESC-derived organoid at 84 DIV is capable of uptake AM1-44 dye. Scale bar, 10 μm. [file Image_2.pdf]

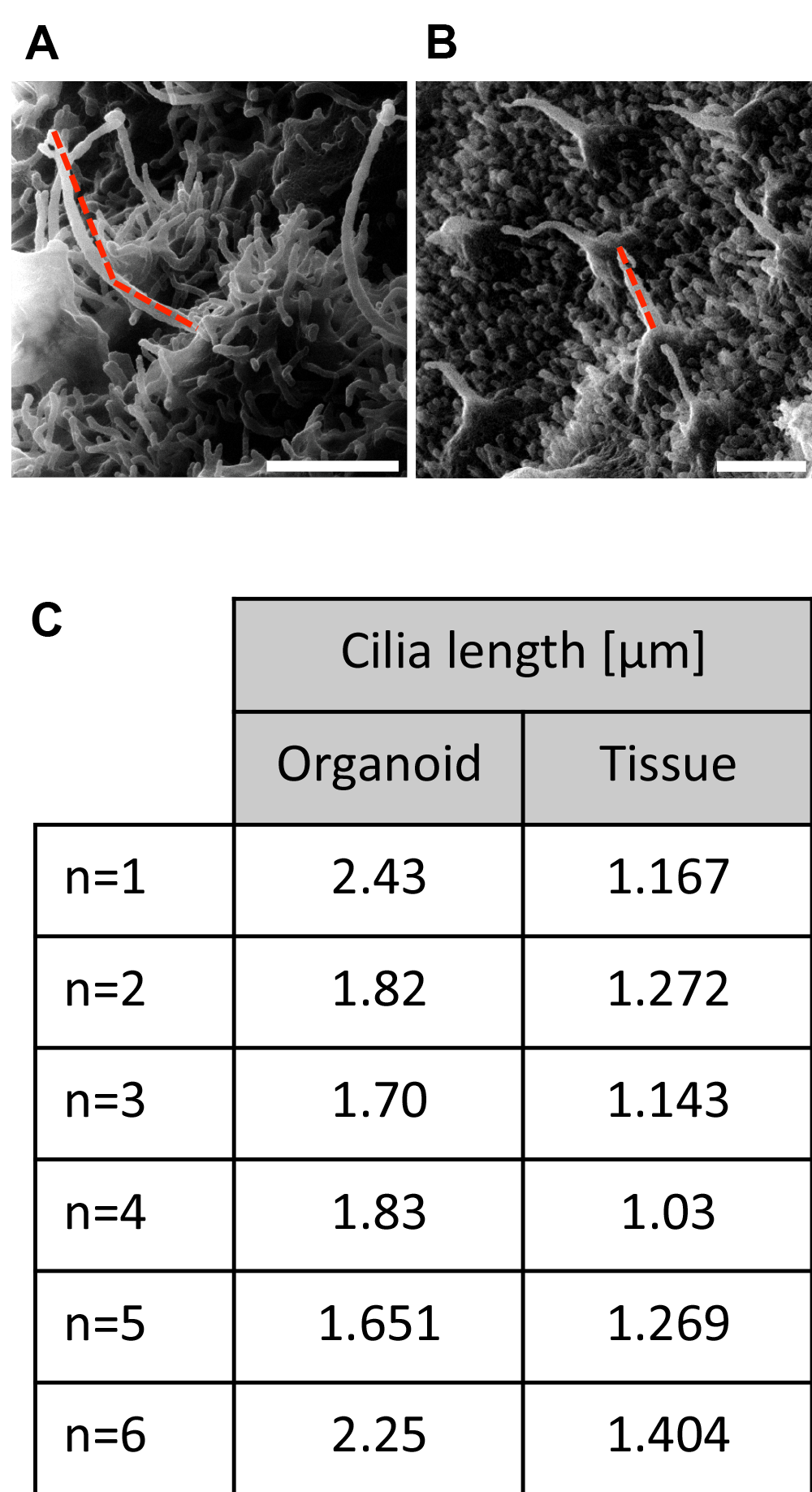

Supplement: Supplementary Figure 3 — Kinocilia length measurements. An example of procedure used for estimating kinocilium length in (A) organoid and (B) fetal tissue. Straight lines were drawn along the cilia (dash lines in red) to estimate the length. (C) Raw data of n = 6 kinocilium measurements each for an organoid and fetal tissue as plotted in Figure 2I. Scale bar, 1 μm. [file Image_3.tif]
