# Supplementary material for: Generation of Vestibular Tissue-Like Organoids From Human Pluripotent Stem Cells Using the Rotary Cell Culture System
Source: Front Cell Dev Biol. 2019 Mar 5;7:25. doi: 10.3389/fcell.2019.00025 (PMC6413170; doi:10.3389/fcell.2019.00025)
Supplement: Supplementary Table 1 — Expression efficiency of ATOH1 in inner ear organoids. ATOH1 expression in organoids derived from hPSC lines (H3 hESC, H9 hESC and 007–5 iPSC lines) in n = 7 biological replicate experiments from 7 to 133 DIV. 58% of organoids showed ATOH1 expression. [file Table_1.pdf]

Supplementary Table 1

	D 7	D 14	D 21	D 28	D 35	D 49	D 112	D 119	D 133
**N=A (H3)**						1/3			
**N=B (H3)**			3/3		0/3		3/3		3/4
**N=C (H9)**			2/2						
**N=D (H9)**			1/1			0/3		4/4	
**N=E (007-5)**	1/2								
**N=F (H9)**	0/3	3/4							
**N=G (007-5)**				1/3					
