# Supplementary material for: Generation of Vestibular Tissue-Like Organoids From Human Pluripotent Stem Cells Using the Rotary Cell Culture System
Source: Front Cell Dev Biol. 2019 Mar 5;7:25. doi: 10.3389/fcell.2019.00025 (PMC6413170; doi:10.3389/fcell.2019.00025)
Supplement: Supplementary Table 2 — Volume of components in each measurement by micro-computed tomography. The table shows the total number of components, as well as the mean, smallest and largest component volumes in each measurement, and the combined total volume of all components in a measurement. The two measurements (scans) of any single sample have not been pooled. [file Table_2.pdf]

Supplementary Table 2

Organoid information	Sample	Measure-ment	Number of components [-]	Total volume of all components [µm³]	Mean component volume [µm³]	Smallest component volume [µm³]	Largest component volume [µm³]
10 weeks	1228	2232	2	0	0	0.00	0
10 weeks	1234	2238	4	4931	1233	105.98	4596.22
12 weeks	1240	2268	9	6179	3390	102.91	3389.95
12 weeks	1240	2269	3	1992	664	102.91	1226.24
12 weeks	1241	2276	8	1280	160	103.94	440.83
14 weeks	1244	2289	40	98977	2501	102.40	58504.70
14 weeks	1244	2290	26	21956	580	110.08	7457.28
14 weeks	1245	2297	1	736	736	735.74	735.74
14 weeks	1245	2298	5	34805	8626	127.49	25843.71
