# Supplementary material for: Generation of Vestibular Tissue-Like Organoids From Human Pluripotent Stem Cells Using the Rotary Cell Culture System
Source: Front Cell Dev Biol. 2019 Mar 5;7:25. doi: 10.3389/fcell.2019.00025 (PMC6413170; doi:10.3389/fcell.2019.00025)
Supplement: Supplementary Table 3 — GMax, V½, and slope values of IV relationship of K+ and Na+ currents in organoid and human hair cells. Unless otherwise specified, all statistical analyses were independent sample t-tests. *p < 0.05, + Mann U Whitney statistical analysis. [file Table_3.pdf]

Supplementary Table 3

		Organoid cells	Human vestibular hair cells
K^+ channel	G_{MAX}	2.25 ± 0.28* n = 6	5.55 ± 0.08* n = 10
	$V_{½}$	2.60 ± 0.62* n = 6	-19.58 ± 0.38* n = 10
	Slope	7.10 ± 0.47 n = 6	6.24 ± 0.35 n = 10
Na^+ channel	G_{MAX}	+10.93 ± 0.45 n = 9	+5.82 ± 0.17 n = 10
	$V_{½}$	-18.45 ± 0.64* n = 9	-36.52 ± 0.62* n = 10
	Slope	5.36 ± 0.60 n = 9	5.32 ± 0.54 n = 10
